# Static cold package for transporting organs for transplants: a validation method and pilot test

**DOI:** 10.1590/1516-3180.2025.2930.29042025

**Published:** 2025-10-27

**Authors:** Sibele Maria Schuantes-Paim, Renata Fabiana Leite, Vanessa Ayres Carneiro Gonçalves, Adriana Aparecida Carbonel, Eliana Cavalari Teraoka, Graciana Maria de Moraes Coutinho, Victor Arayama Cruz, Manuel de Jesus Simões, Andre Ibrahim David, Murched Omar Taha, Janine Schirmer, Bartira de Aguiar Roza

**Affiliations:** I PhD candidate, Escola Paulista de Enfermagem (EPE), Universidade Federal de São Paulo (Unifesp), São Paulo (SP), Brazil.; II PhD candidate, Escola Paulista de Enfermagem (EPE), Universidade Federal de São Paulo (Unifesp), São Paulo (SP), Brazil.; III MA student, Escola Paulista de Enfermagem (EPE), Universidade Federal de São Paulo (Unifesp), São Paulo (SP), Brazil.; IV Coordinator of Clinical Research, Hospital das Clínicas, Faculdade de Medicina, Universidade de São Paulo (FMUSP), São Paulo (SP), Brazil.; V Escola Paulista de Enfermagem (EPE), Universidade Federal de São Paulo (Unifesp), São Paulo (SP), Brazil.; VI Escola Paulista de Enfermagem (EPE), Universidade Federal de São Paulo (Unifesp), São Paulo (SP), Brazil.; VII MA student, Escola Paulista de Enfermagem (EPE), Universidade Federal de São Paulo (Unifesp), São Paulo (SP), Brazil.; VIII Professor, Laboratory of Structural and Molecular Gynecology, Universidade de São Paulo (USP), São Paulo (SP), Brazil.; IX Professor, Escola Paulista de Medicina (EPM), Universidade Federal de São Paulo (Unifesp), São Paulo (SP), Brazil.; X Professor, Escola Paulista de Medicina (EPM), Universidade Federal de São Paulo (Unifesp), São Paulo (SP), Brazil.; XI Professor, Escola Paulista de Enfermagem (EPE), Universidade Federal de São Paulo (Unifesp), São Paulo (SP), Brazil.; XII Professor, Escola Paulista de Enfermagem (EPE), Universidade Federal de São Paulo (Unifesp), São Paulo (SP), Brazil.

**Keywords:** Product packaging, Transplants, Validation study, Static cold package, Transport, Logistics, Organ transplantation, Transplantation, Validation method

## Abstract

**BACKGROUND:**

Logistic and temperature challenges contribute to organ loss during transplantation. Ensuring the safety of static cold packaging for organ transport is essential to improve patient access to transplants. This study aimed to verify a method for validating the packaging used to transport organs for transplantation.

**DESIGN AND SETTING:**

Validation study and pilot test using experimental surgery on porcine organs.

**METHODS:**

Data collection considered the variables related to organ integrity before and after transportation, including temperature (measured thrice with three instruments per organ), macroscopic evaluation (based on photographic and observational assessments), histology (structural analysis of the collected samples), and packaging contamination (triple-swab sampling for microorganism growth). Data analysis was performed using descriptive statistics, visual assessment, histological processing, and microbiological evaluation.

**RESULTS:**

By the end of transportation, all the organs reached the ideal temperature range for transplantation. The similarity in swine weight and size enabled macroscopic comparisons. Histological analysis revealed no significant injuries or morphological changes. Regarding packaging, environmental microorganisms predominate, with sustainable post-transport differences.

**CONCLUSION:**

The method developed to validate the package used for transporting organs for transplantation was successfully verified. Furthermore, this method addresses the existing gap in the process of documenting a robust validation method for packaging intended for organ transportation.

## INTRODUCTION

 Organ donation and transplantation in Brazil are among the world’s most advanced, with approximately 25,000 transplants performed by 2023. However, nearly 60,000 people remained on waiting lists.^1^ The leading cause of organ loss was family refusal to donate (42%), followed by clinical contraindications (17%) and logistical issues (15%).^1^ These challenges highlight the need for strategies to improve donation rates and optimize organ transportation.^2-3^

 Brazil’s vast territory and extreme temperature variations complicate its transportation logistics. Organs must be kept between 2°C and 8°C,^4-7^ while some regions exceed 45°C.^8^ A 2023 report noted a 3°C temperature increase, which further affects safe transport.^9^ Given these constraints, evaluating the safety and efficacy of organ transport packaging is crucial. 

 A literature search revealed that no comprehensive method for validating static cold packages to ensure organ integrity. To address this gap, we developed a robust, reliable, and replicable validation method for organ transport packaging. 

 This study aimed to verify a method for validating the packaging used for transporting organs for transplantation. 

## METHODS

 This was a validation study and pilot test for experimental surgery using porcine organs. This experimental study assessed the variables before and after the transportation of porcine organs (Landrace swine). Four categories were analyzed to evaluate organ integrity and packaging: temperature, macroscopic appearance, histology, and microbiological growth. This research report refers to the development of two pilot tests of the method conducted in November (P1) and December (P2) of 2022 and describes all modifications in the method until the final protocol based on the results. 

### Packaging regulations

 In accordance with Brazilian legislation, each organ must be packaged in three sterile plastic bags. The first bag contains the organ and preservation solution, the second bag encloses the first bag and the cold physiological solution, and the third bag contains both the previous bags. All these packages are placed in a domestic plastic cooler with ice for transportation (static cold).^4^

### Animals

 Human research is not feasible due to ethical constraints, the need for thorough organ analysis, and the requirement for a controlled environment, which is impractical in clinical transplantation. Despite its limitations, hypothermic static cold storage with ice remains the preferred global method for organ transport, yielding positive outcomes despite the gaps in data on organ integrity. 

 To address these challenges, we used porcine organs from Landrace swine, given their anatomical similarity to human organs and their role in xenotransplantation trials.^10^ This pilot test involved two female Landrace pigs (≈25 kg) from a farm affiliated with the Universidade Federal de São Paulo (Unifesp). The animals received proper care before transportation and were housed in a ventilated wooden transport box under veterinary supervision upon arrival. 

### Anesthesia, monitoring, and surgical procedure

 Animals received pre-anesthetic medication with ketamine (15 mg/kg) and midazolam maleate (0.2 mg/kg) intramuscularly, followed by intravenous propofol (7 mg/kg) after 3 minutes. They were then intubated with a 7.0 orotracheal tube with a cuff and maintained under inhaled anesthesia (1.5–2% isoflurane) for 4 hours. Analgesia was provided with fentanyl citrate (2.5 mg/kg) at surgery initiation and every 2 hours, with propofol, as needed. Electrodes and oximeters were used for continuous monitoring. 

 After thoracoabdominal incision and aortic clamping, the animals were declared dead and cold ischemia was initiated. The heart, liver, pancreas, and kidneys were retrieved under the supervision of physicians and veterinarians to ensure minimal suffering. The animals remained on the surgical table throughout the procedure, and at the end of the procedure, all organs were reintroduced into the cavity. The animals were then stored in a freezer until collection by São Paulo city’s specialized service. The experiment was conducted at the Laboratory of Operative Technique and Experimental Surgery, Unifesp, under the supervision of two physicians, a veterinarian, and a veterinary assistant. 

### Data collection

**Figure 1:**
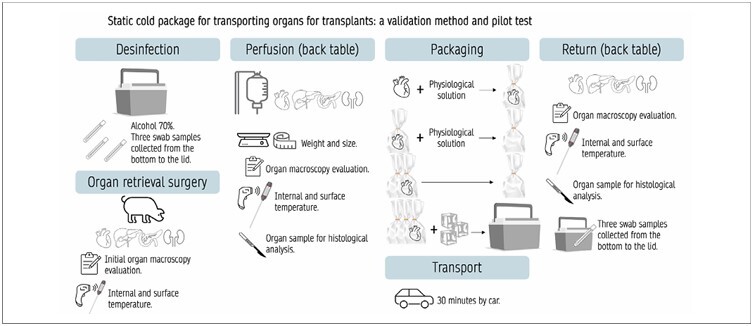
Data collection method.

 Data were collected at various time points throughout the experiment. After organ retrieval, the first collection of macroscopic aspects was performed. Each organ was then placed on a back table where other researchers, nurses, and undergraduate students proceeded with perfusion and continued with data collection and packaging. Once packaging was complete, the organs were transported by car through a simulated circuit. After their return, all data collection steps were repeated (Figure 1). 

### Temperature

 Data related to the "temperature" variable was collected at different stages: H0: as soon as the organ was retrieved from the animal cavity; H1: after perfusion on the back table, before packaging; H2: after transportation. 

 Temperature measurements of the pancreas were not performed at time point H1 because, after the initial data collection, the pancreas did not undergo perfusion and was immediately placed in the packaging. 

 In the first surgery (P1), only one thermometer was used to measure the internal temperature. However, in the second surgery (P2), we employed an additional thermometer to obtain the average internal temperature for each organ and an infrared thermometer to measure the surface temperature. This paper presents the values from the internal thermometers for the first surgery and the averages from the second surgery derived from the internal thermometers, in addition to the superficial readings. 

### Organ macroscopy

 Organ macroscopic data were recorded using study-specific forms based on macroscopic evaluation studies.^11-14^ Each organ had a unique form, which was completed at retrieval, after perfusion, and post-transport by physicians and nurses. Evaluations included visual inspection with responses in "yes/no" format or on a 1–10 scale. 

 Weight was measured using a scale and size was measured with a ruler. From the second experiment onward, each organ was photographed immediately after perfusion and post-transport to document the changes. 

### Histology

 The tissue samples were collected after perfusion and post-transport unpacking. Each sample was fixed in 4% paraformaldehyde (phosphate buffer) for 24 hours, then processed through dehydration in graded alcohol, diaphanization with xylene, and paraffin impregnation at 60°C. 

 The samples were then embedded for histological cross-sectional analysis. Using a Minot-type microtome, 4 μm sections were cut with 50 μm spacing and mounted on slides for hematoxylin and eosin (H&E) staining (histomorphometric analysis). 

### Packaging

 Before surgery, the transport packaging was disinfected by applying 70% alcohol to the interior, followed by wiping the internal walls and lid with disposable paper in unilateral motion. This process was repeated twice and applied to the external walls. 

 Three swab samples were collected from the bottom to the lid, following microbiological sampling standards (sample, test, and control). After transportation, the researchers removed the ice and repeated the swab collection. Initially, samples were collected from each internal wall. However, after the first analysis, the protocol was standardized to three swabs from the entire packaging. 

## DATA ANALYSIS

### Temperature

 Temperature data were subjected to quantitative analysis using descriptive statistics, specifically calculating the average temperature. 

### Macroscopy

 Pre-transport assessments included tissue/parenchymal quality, overall organ quality, perfusate appearance after 5 min, and perfusion quality (parenchymal discoloration). Post-transport evaluations considered tissue/parenchyma quality, overall organ quality, and perfusion quality. 

 Overall organ quality was rated on a scale of 1–10, with 10 being the highest. On average, the stability indicated no change, an increase suggested improvement, and a decrease indicated deterioration. Abnormalities including lesions were documented before and after transport for comparison. Parenchymal coloration was assessed for homogeneity (ideal) or heterogeneity (potential perfusion issues) in both phases. 

### Histology

 Histological samples were analyzed using H&E staining, enabling the differentiation of basophilic (stained by hematoxylin) and acidophilic or eosinophilic (stained by eosin) structures. This technique allows the observation of images via light microscopy using an optical microscope. In H&E staining, cell nuclei appear as blue-purple shades owing to their basophilic nature, as the cell nucleus contains deoxyribonucleic acid (DNA), which attracts hematoxylin, a basic dye. Conversely, the cell cytoplasm, with its more basic character, was stained pinkish-red by eosin staining. Some regions of the cytoplasm appear bluish because of the presence of ribonucleic acid (RNA), which is stained with hematoxylin. Organ structures were carefully observed and analyzed to identify morphological alterations. 

### Microbiologic samples

 The laboratory provided a report on microbial growth within the packaging after processing the samples using matrix-assisted laser desorption ionization time-of-flight mass spectrometry (MALDI-TOF MS). Data analysis relied primarily on the measurement of colony-forming units (CFU). 

### Ethics statement

 This study was conducted in strict accordance with the recommendations of the Guide for the Care and Use of Laboratory Animals of the National Health Authority. The study protocol was approved by the Committee on the Ethics of Animal Experiments of the Universidade Federal de São Paulo (Unifesp; Protocol No. 4197081221). All efforts were made to minimize animal suffering. 

## RESULTS

### Temperature

 Because the surgery replicated human organ procurement in swine, temperature data were collected at critical moments when fluctuations could occur. The duration for which each organ remained in the packaging from the initial (H1) to the final (H2) temperature measurements is detailed in Table 1. 

**Table 1:** Duration of each organ storage in minutes within the packaging (P1 + P2)

**Organs**	**P1 (min)**	**P2 (min)**	**Average (min)**
Heart	163	110	131,5
Liver	89	114	101,5
Pancreas	86	115	100,5
Right kidney	75	99	87
Left kidney	71	96	83,5
**Total**			**100,8**

**Figure 2:**
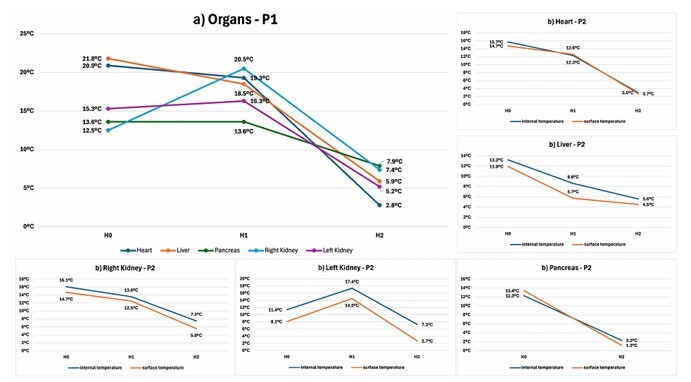
Temperature patterns of each organ in the surgeries (P1 and P2). a) Temperature patterns obtained from the internal thermometers of each organ during the first surgery (P1). b) Patterns obtained from the average of the internal thermometers used in the second surgery (P2) compared with the surface temperature measurements.

**Figure 3:**
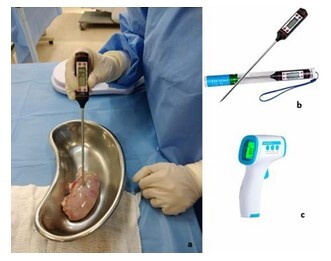
Temperature measurement process and thermometers. a) Measurement methods. b) Thermometer used to assess the internal temperature: after the insertion, researchers keep the thermometer in the parenchyma until the temperature mark stabilizes. c) Infrared thermometer: researchers keep the thermometer at a distance of 2 cm for 2–3 s according to the manufacturer’s guidance provided in the product manual.

Figure 2 illustrates all the temperature measurements from the two experiments. The temperature patterns obtained from the internal thermometers of each organ during the first surgery (P1) are shown in Figure 2a. The patterns obtained from the average of the internal thermometers used in the second surgery (P2) compared with the surface temperature measurements are presented in Figure 2b for each organ. Figure 3 shows the thermometers used and the temperature measurement process. 

### Organ macroscopy

 The average weight in the two experiments for each organ was 158,5 g for the hearts, 607 g livers, 64 g pancreas, and 69,2 g for the kidneys. Table 2 presents the qualitative macroscopic evaluation data for each organ (heart, liver, pancreas, right kidney, and left kidney) in both pilot experiments (P1 + P2), as assessed using the forms developed. 

**Table 2:** Macroscopic evaluation data in each pilot test (P1 + P2)

**Heart**	**Pre transport**		**Post transport**		**Evaluation**	
	**P1**	**P2**	**P1**	**P2**	**P1**	**P2**
Abnormality of the organ	None	None	None	None	No change	No change
Tissue quality	10	10	10	10	No change	No change
Overall quality	10	10	10	10	No change	No change
Perfusate appearance on the bench after 5 minutes	8	7	–	–	Improved	Improved
Perfusion quality (degree of parenchymal discoloration)	8	6	10	10		
Homogeneous coloration of the parenchyma after perfusion	Yes	No	Yes	Yes	No change	Improved
Liver	Pre transport		Post transport		Evaluation	
	P1	P2	P1	P2	P1	P2
Abnormality of the organ	None	None	None	None	No change	No change
Parenchyma quality	7	10	5	9	Worsened	Worsened
Overall quality	7	10	6	9	Worsened	Worsened
Perfusate appearance on the bench after 5 minutes	6	8	–	–	Worsened	Improved
Perfusion quality (degree of parenchymal discoloration)	7	9	6	10		
Homogeneous coloration of the parenchyma after perfusion	No	Yes	No	Yes	Improved	Improved
Pancreas	Pre transport		Post transport		Evaluation	
	P1	P2	P1	P2	P1	P2
Color	Pink-gray	Yellow	Pink	Milky white	Improved	Improved
Tissue injury	None	None	None	None	No change	No change
Calcification	None	None	None	None	No change	No change
Stiffness	None	None	None	None	No change	No change
Hematoma	No	No	No	No	No change	No change
Edema	No	No	No	No	No change	No change
Right kidney	Pre transport		Post transport		Evaluation	
	P1	P2	P1	P2	P1	P2
Abnormality of the organ	None	None	None	None	No change	No change
Parenchyma quality	10	10	8	10	Worsened	No change
Overall quality	10	10	9	10	Worsened	No change
Perfusate appearance on the bench after 5 minutes	9	10	–	–	Worsened	Improved
Perfusion quality (degree of parenchymal discoloration)	10	9	9	10		
Homogeneous coloration of the parenchyma after perfusion	Yes	Yes	No	Yes	Worsened	No change
Left kidney	Pre transport		Post transport		Evaluation	
	P1	P2	P1	P2	P1	P2
Abnormality of the organ	None	None	None	None	No change	No change
Parenchyma quality	10	10	8	10	Worsened	No change
Overall quality	10	10	9	10	Worsened	No change
Perfusate appearance on the bench after 5 minutes	8	9	–	–	Worsened	No change
Perfusion quality (degree of parenchymal discoloration)	9	8	9	10	No change	Improved
Homogeneous coloration of the parenchyma after perfusion	No	Yes	No	Yes	No change	No change

Source: Dabare et al.;^13^ Dare et al.;^12^ Kulu et al.;^14^ Tierie et al.^11^

**Figure 4:**
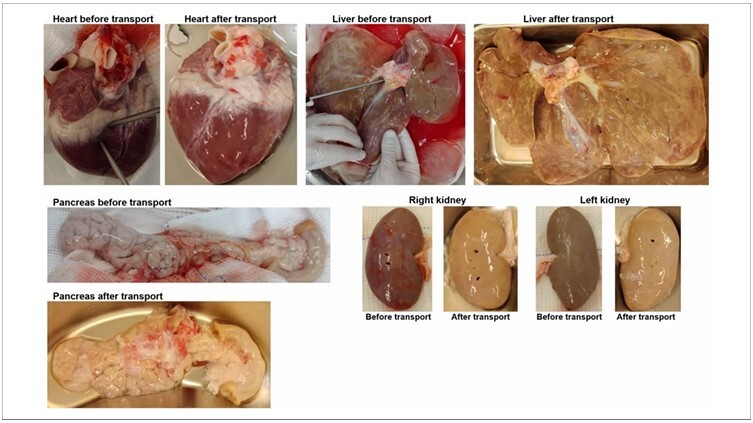
Photographs of organs after perfusion, before and after transportation, in P2.

 Comparative photographs of the organs after perfusion before and after transportation in P2 are shown in Figure 4. 

### Histology

**Figure 5:**
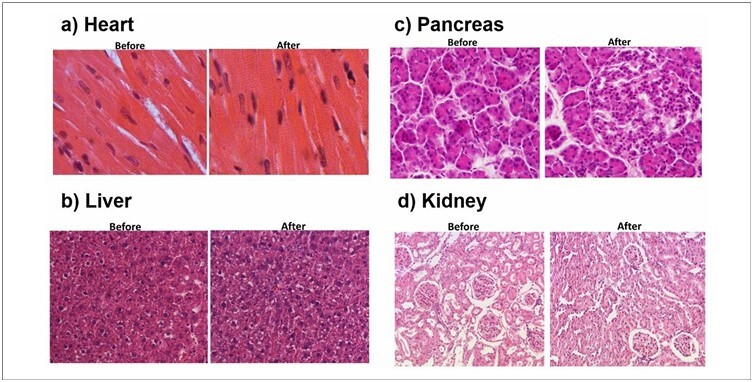
Photomicrograph showing sections of pig hearts, livers, pancreas, and kidneys before and after transportation. 20× HE.

 The morphological structures of the organs were examined. Each organ was analyzed separately (Figure 5). 

 In the heart (Figure 5a), the myocardial morphology of both ventricles was similar across the study groups. The myocardium consists of small elongated cardiac fibers with one or two central nuclei that display transverse striations surrounded by small capillaries. The endocardium was lined by simple squamous epithelium with a small amount of connective tissue beneath it, whereas the epicardium showed loose connective tissue with some adipose cells. 

 The hepatic parenchyma exhibits integrity in its histological structure and is composed of a high concentration of hepatocytes arranged in cords that converge towards the central lobular vein, forming approximately hexagonal geometric figures. Between the cords of hepatocytes, hepatic sinusoids were observed, lined by endothelial cells with nuclei of various shapes, ranging from elongated to spherical, and usually heterochromatic. Hepatocytes are voluminous polyhedral cells with one or two centrally positioned spherical nuclei rich in chromatin and well-defined nucleoli. 

 The cytoplasm was not homogeneous and displayed areas with basophilic and eosinophilic characteristics. Red blood cells were identified within the sinusoidal capillaries. Within the portal space, at least one branch of the portal vein, hepatic artery, or bile duct can be identified. Connective tissue cells and rare collagen fibers surrounded these structures (Figure 5b). 

 The pancreas (Figure 5c) is a mixed gland with both exocrine and endocrine components. The exocrine portion is formed by numerous acini, among which we identified endocrine portions called the pancreatic or Langerhans islets. The acini are globular structures composed of prismatic cells containing spherical nuclei located in the middle of the cells or slightly shifted towards the basal region. The pancreatic islets are formed from cords containing cells with spherical nuclei. 

 The kidneys (Figure 5d) showed preserved renal parenchyma, especially in the cortical region, with intact glomeruli, and proximal and distal convoluted tubules. The renal corpuscles, including the intact Bowman’s capsule with a visible urinary space and glomeruli composed of endothelial-lined capillaries, podocytes, and mesangial cells, showed no visible histological alterations. Most of the renal cortex comprises glomeruli and tubules. 

### Microbiological growth

 As described in the Methods section, data collection was standardized after the second surgery. In this paper, the results obtained following the data collection pattern at P2 are presented. Table 3 shows the microorganisms found in the packages, comparing pre- and post-transport results. 

**Table 3:** Standardized protocol for sample collection and results obtained in the second pilot experiment (P2), considering samples collected pre-transport (pre) and post-transport (post)

**Transported organ**	**Sample 1**		**Sample 2**		**Sample 3**	
	**Pre**	**Post**	**Pre**	**Post**	**Pre**	**Post**
Liver	None	None	None	None	None	None
Pancreas	None	None	None	None	None	None
Heart	*Micrococcus luteus* (90CFU) *Staphylococcus warneri* (20CFU) *Penicillium* spp.	None	*Micrococcus luteus* (40CFU) *Staphylococcus warneri* (10CFU)	None	*Micrococcus luteus* (20CFU) *Staphylococcus warneri* (5CFU)	None
Kidney	None	None	None	None	None	None

CFU: Colony Forming Unit

## DISCUSSION

### Temperature

 Regarding the temperature range, it’s important to note that while the typical range cited in literature falls between 2°C and 8°C, there are varying ranges reported. Factors such as the preservation solution and the time between organ retrieval and packaging may contribute to this variation. However, studies examining organ integrity, transplant outcomes, and organ transportation have shown a range of temperatures from 0°C to 10°C.^5-7,15-20^ In this study, we have chosen to use the most common temperature range as our reference point for analyzing the impact of temperature on organ integrity. 

 During the first surgery, the organs tended to warm after perfusion. To improve the outcomes, we introduced additional variables in the second experiment (P2) to better control the data collection. These included maintaining a stable room temperature (18°C), using three liters of physiological solution within the animals’ cavities, tightly regulating temperature with a freezer, and employing smaller pieces of crushed ice to lower cavity temperatures. 

 These experiments did not use preservation solutions but instead used physiological solutions. We emphasize the need for future research to incorporate appropriate preservation solutions to enhance the resemblance to human surgical conditions and improve overall organ quality. 

### Macroscopy

 In the context of organ macroscopy, the similarity in the weight and size of the pigs in both experiments (P1 and P2) facilitated a comparative analysis. The qualitative interpretation of each organ using the data collection instrument revealed substantial differences, particularly in the case of the liver, before and after transportation. This discrepancy underscores the susceptibility of the liver to transportation-induced stress.^21^ Other organs, despite being transported, demonstrate good color and perfusion characteristics. Although the evaluation was qualitative and subject to the researcher’s judgment, it may serve as a valuable tool to be validated for application in human scenarios. 

 The amalgamation of data from instruments and photographs aids in understanding the rationale behind these classifications. Moreover, it provided visual evidence to facilitate data comparison. Photographs serve as a resource for future comparative studies by establishing defined patterns.^22^

### Histology

 The assessment of organ integrity, a combination of macroscopic analysis in two distinct ways, and histological analysis helped identify areas of damage during transportation. These analyses shed light on the changes that the organs undergo during transportation. Histological analysis indicated that the examined groups did not exhibit significant injury or morphological alterations. However, it has become evident that structural analysis alone may not reveal all the damage, prompting consideration of the inclusion of immunohistochemical analysis in future experiments.^23^

### Microbiology

 Specific discussions of microorganisms have provided interesting insights. *Micrococcus luteus* is commonly found in natural environments, such as soil and water resources, and is considered a normal inhabitant of human skin and oropharynx mucosa.^24-25^ *Staphylococcus warneri* is part of the normal skin flora, particularly in the nares, head, legs, and arms.^26^ *Corynebacterium imitans* is present within the human oral cavity and airways,^27^ while *Penicillium* spp. is one of the most widespread fungi in various environments.^28^

 The presence of environmental microorganisms in the pre-transport phase, despite following cleaning and disinfection protocols, suggests that microorganisms may proliferate even with proper protocol adherence. This highlights the need for maintaining high standards of disinfection. Although packaging is not sterile, some growth is expected; however, the use of ice effectively inhibits microorganism proliferation. 

### Comparison with prior work

 Previous studies on organ transportation have predominantly focused on examining individual variables associated with organ integrity. Recent research efforts have been directed towards developing new products and packages aimed at enhancing the safety of organ transportation for transplantation. Conversely, previous studies have concentrated on the feasibility of transporting organs on ice.^29-34^ To the best of our knowledge, this is the first study to specifically investigate organ transport for transplantation, encompassing the analysis of four variables (temperature, organ macroscopy, histology, and microbiological growth within the package) to ascertain the safety of such transportation method. 

### Limitations

 The first limitation pertains to the duration for which each organ remains in the package. Although the analysis yielded satisfactory results, it required an average time of 100.8 minutes. Another limitation is the use of a limited number of animals. This limitation was implemented to minimize suffering; hence, we restricted the pilot test to two animals. Finally, this report presents a pilot test aimed at validating the method and underscores the necessity for replicability by incorporating diverse scenarios to substantiate the concept. 

## CONCLUSION

 After interpreting the data and conducting experiments, this pilot test successfully verified the developed method for validating the packaging used to transport organs for transplantation. 

 Considering the variables under investigation, it is evident that temperature measurement methods are effective tools for monitoring the temperature patterns in the studied organs. The combination of macroscopic and histological analyses and photography offers comprehensive insights into the condition of the examined organs. The inclusion of standardized histological analyses provides valuable information about organ integrity, and standardized microbiological analyses effectively demonstrate microbiological growth. 

 The method developed in this study can serve as a valuable tool for validating packaging used in the safe transportation of organs for transplantation. This research is significant because it addresses the existing gap in documenting a robust validation method for packaging intended for organ transportation. By integrating complementary data and scrutinizing variables that directly affect organ integrity and package safety, this method effectively ensures product reliability. 
